# Severe Frostbite Due to Extreme Altitude Climbing in South America: A Case Report

**DOI:** 10.7759/cureus.27771

**Published:** 2022-08-08

**Authors:** Alejandro J Quiroz Alfaro, Iván Javier Rodríguez Acosta, José D Cardona, Andrés Felipe Herrera Ortiz

**Affiliations:** 1 Medicina y ciencias de la salud, Universidad Colegio Mayor de Nuestra Señora del Rosario, Bogotá D.C, COL; 2 Department of Radiology, Fundacion Santa Fe de Bogota, Bogotá D.C, COL; 3 Department of Radiology, Universidad El Bosque, Bogotá D.C, COL

**Keywords:** cold injury, south america, high altitude, mountaineer, amputation

## Abstract

Frostbite is a tissue injury secondary to freezing that can currently be categorized using two schemes (Cauchy and clinical scheme). However, we present a fourth-degree frostbite case with overlapping features between both classifications, generating difficulty in categorizing it using either. We wanted to raise awareness of such an atypical presentation and propose employing both classifications to define the extent and compromise of frostbite more appropriately.

## Introduction

Frostbite (FB) is defined as a tissue injury secondary to freezing. Prefreeze, freeze-thaw, vascular stasis, and late ischemic are its four overlapping pathological phases [1]. FB severity can be classified into four degrees: first-degree causes numbness, erythema, and a plaque in the injured area, without tissue infarction; second-degree causes superficial skin blistering; third-degree involves deeper skin layers, causing hemorrhagic blisters; fourth-degree involves muscle and bone [1]. FB is a rare diagnosis; its incidence in adults in the United States was 0.95 per 100,000 individuals. However, it is exceptionally incapacitating, in severe cases, leading to severe dysfunction and amputations [2]. We present an unusual case of a patient who consulted belatedly, presenting fourth-degree FB, compromising eight of his ten fingertips and distal phalanges, requiring amputation.

## Case presentation

A 35-year-old previously healthy male patient arrived at the ED due to blue-gray discoloration of his fingers. One week earlier, the patient had been mountaineering gloveless in "La Sierra Nevada del Cocuy" (Colombia), of which maximum height can go up to 5380 meters above sea level and has a mean temperature ranging from 24 ºC to -3 ºC (75.2 to 26.6 °F).

A few hours after descending, the patient noticed painless blue-gray discoloration and hemorrhagic blisters on his fingertips that he thought would improve with rewarming with warm water. However, a week afterward, after noticing a persisting loss of sensation and blue-gray discoloration of the fingertips, eventually turning black, the patient decided to consult. 

**Figure 1:**
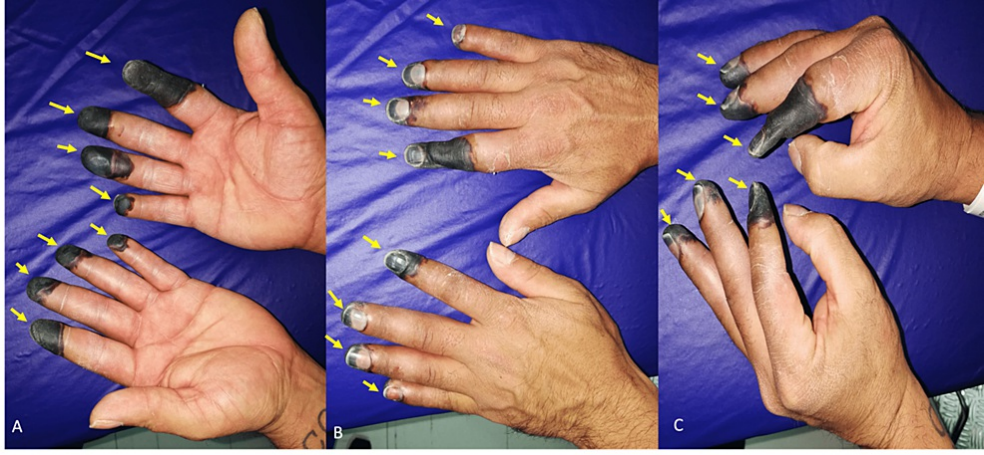
(A, B, and C) Fourth-degree frostbite of the second, third, fourth, and fifth fingers of both hands (yellow arrows).

The patient denied smoking and any other relevant medical history. During the physical examination, he had normal vital signs, absent capillary refill, and distal perfusion with necrosis of the fingertips and distal phalanges of his second to fifth fingers bilaterally, with more extensive involvement of the right index extending to the middle phalanx (Figure 1). The rest of the physical exploration was unremarkable; therefore, the patient was diagnosed with FB.

Color Doppler of the hands showed a lack of arterial and venous flow with an extension correlating to the necrotic areas evidenced on the physical examination. Based on the absence of blood flow and the elapsed time, the distal phalanges of the second to fifth fingers were amputated bilaterally; the right index required amputation extending to the middle phalanx. 

One week afterward, the patient recovered from surgery without complications and remained in physical rehabilitation.

## Discussion

Different treatment options have been proposed for FB. Amongst them are general measures like pain control, the elevation of the affected extremity, excision of necrotic tissue, debridement of tense blisters to increase motion, and thrombolysis in patients with a warm-ischemia time of fewer than 24 hours [3].

Thrombolysis has shown clear evidence of reducing amputations secondary to FB. A systematic literature review with 17 studies reported an overall digit salvage rate of 81.2% [4].

In our case, neither were general measures implemented because the patient showed up with dry, painless necrotic areas a week after rewarming the affected extremities, nor was thrombolytic therapy recommended since the elapsed warm-ischemia time was more than 24 hours. A complete absence of blood flow evidenced by the color Doppler made amputation the only possible treatment option.

It is important to note that since our patient had fourth-degree FB, compromising bone, he most likely had impaired sensation and anesthetic areas from the beginning. Therefore, we consider the absence of pain the main factor preventing him from consulting earlier.

Usually, one of the two four-level classification schemes is used to determine the FB severity (Table 1) [3].

**Table 1:** Frostbite classification schemes. Adapted from Sheridan RL et al. [3].

Classification scheme	Classification level			
Clinical scheme	First Degree	Second Degree	Third Degree	Fourth Degree
Depth of injury	Superficial, may include nonfrozen cold injury	Within the dermis	Full thickness skin	Tissue beneath skin, including muscle tendon and bone
Initial findings	Reduced sensation, erythema, and burning after rewarming	Clear blistering with later sloughing of necrotic skin, pain with rewarming	Blue-gray skin discoloration; blisters that are clear, hemorrhagic, or both; pain with rewarming	Blue-gray skin discoloration, no pain with rewarming
Sequelae	None	Lasting cold sensitivity may develop	Full thickness skin wounds, damage to growth plates in children	Full-thickness skin wounds, necrosis of underlying bone and deep tissue
Cauchy scheme	Grade 1	Grade 2	Grade 3	Grade 4
Extent of initial lesion	No lesion	Lesion on distal phalanx	Lesion on middle proximal phalanx	Lesion on carpal or tarsal area
Bone scanning on day 2	Scanning unnecessary	Hypofixation of radiotracer	Absence of radiotracer uptake in digit	Absence of radiotracer uptake in carpal or tarsal area
Blisters on day 2	None	Clear blisters	Hemorrhagic blisters	Hemorrhagic blisters
Prognosis on day 2	No sequelae	Tissue amputation	Bone amputation of digit	Bone amputation of limb

Both classifications should be used to more precisely assess the extent, depth, severity, and possible compromise of the affected extremity. As evidenced in our case, the majority of the affected fingers, except for the right index, could have been classified as Cauchy grade 2. Nonetheless, grade 2 usually requires tissue debridement as treatment rather than amputation [5]; since our patient had blood flow compromise and extensive necrosis, amputation was needed.

It is pertinent to point out that the Cauchy scheme is most helpful in classifying and predicting amputation in FB secondary to extreme temperatures, with a warm-ischemia time of no more than 48 hours and affected areas located mainly in the hands and feet [5]. Making it less useful when classifying FB located in different areas of the body, especially if the causal mechanism was accidentally self-inflicted or iatrogenic (ice packs, cooling blankets) [6-9]. The clinical scheme is more widely used for accidentally self-inflicted or iatrogenic FB. We found four case reports [6-9] where the FB was classified from second to fourth degree using the clinical scheme, affecting from 1% to 10% of total body surface area, requiring treatments ranging from topical ointments to tissue debridement and skin grafting. However, amputation was unnecessary in these cases. This suggests that the clinical scheme might not help predict amputation in accidentally self-inflicted or iatrogenic FB.

The clinical scheme only determines depth, not the extension; however, the third and fourth degrees are clinically very similar, differing only in the appearance of pain while rewarming on the third degree [3]. Since the clinical scheme can have overlapping features, some patients may still have blue-gray skin discoloration and blisters without blood flow or deep tissue compromise like bone [6].

In our experience, some cases with local compromise, especially secondary to the usage of ice packs or dry ice on swollen areas, can have FB of delimited areas like the middle phalanx of a specific finger, making the classification more difficult using only the Cauchy scheme.

In the future, a more robust classification scheme that could more precisely determine the extension, depth, severity, and predicting amputation independently of the location or causal mechanism of FB should be implemented. Meanwhile, we suggest using both the existing classifications.

## Conclusions

Both clinical and Cauchy classifications should be used to assess the FB extent and profundity appropriately since, as evidenced, the Cauchy classification more precisely defines the extension. In contrast, the clinical scheme more precisely defines depth, generating the necessity to employ both to determine severity more accurately. It is relevant to raise awareness that patients with severe FB can consult belatedly secondary to impaired sensation.
